# *LIMD2* is a Prognostic and Predictive Marker in Patients With Esophageal Cancer Based on a ceRNA Network Analysis

**DOI:** 10.3389/fgene.2021.774432

**Published:** 2021-11-18

**Authors:** Yuanmei Chen, Xinyi Huang, Kunshou Zhu, Changkun Li, Haiyan Peng, Lin Chen, Zhengrong Huang, Yangfan Zhang, Guibin Weng, Tianya Xiao, Junqiang Chen, Yuanji Xu

**Affiliations:** ^1^ Department of Thoracic Surgery, Fujian Medical University Cancer Hospital, Fujian Cancer Hospital, Fuzhou, China; ^2^ Department of Radiation Oncology, Fujian Medical University Cancer Hospital, Fujian Cancer Hospital, Fuzhou, China; ^3^ Fujian Key Laboratory of Innate Immune Biology, Biomedical Research Center of South China, Fujian Normal University Qishan Campus, Fuzhou, China; ^4^ Department of Integrative Traditional Chinese and Western Medicine, Fujian Medical University Cancer Hospital, Fujian Cancer Hospital, Fuzhou, China

**Keywords:** esophageal cancer, competing endogenous RNA, LIM domain containing, prognostic, predictive

## Abstract

Globally, esophageal cancer (ECA) is the seventh most common cancer and sixth most common cause of cancer-associated mortality. However, there are no reliable prognostic and predictive molecular markers for ECA; in addition, the pathogenesis of ECA is not fully elucidated. The expressions of circular RNAs (circRNAs), microRNAs (miRNAs), and messenger RNAs (mRNAs) of ECA and control groups were obtained from the RNA-sequencing (RNA-seq) data of our hospital, the Gene Expression Omnibus (GEO), and The Cancer Genome Atlas (TCGA) datasets. Analyses of differentially expressed genes, the circRNA–miRNA–mRNA–competing endogenous RNA (ceRNA) network, and functional/pathway enrichment were conducted. The key targets in the ceRNA network that showed significant results in survival Cox regression analyses were selected. Furthermore, analyses of immune infiltration and autophagy genes related to the key targets were performed. Seven circRNAs, 22 miRNAs, and 34 mRNAs were identified as vital genes in ECA; the nuclear factor-κ-gene binding (NF-κB) and phosphatidylinositol-3 kinase/protein kinase B (PI3K-Akt) signaling were identified as the most enriched pathways. In addition, the LIM domain containing 2 (*LIMD2*) was an independent predictor of prognosis in ECA patients and closely associated with immunity and autophagy. Moreover, quantitative reverse-transcription polymerase chain reaction (qRT-PCR) revealed significant upregulation of *LIMD2* expression in ECA tissues. ECA may be closely correlated with NF-κB and PI3K/Akt signaling. In addition, *LIMD2* could be a potential prognostic and predictive marker of ECA.

## Introduction

Esophageal cancer (ECA), including esophageal squamous cell carcinoma (ESCC) and esophageal adenocarcinoma (EAC), is the seventh most common malignant tumor in the world and the sixth most common primary cause of tumor-associated death, especially in Asia (Bray et al., 2018). In 2018, approximately 572,034 new ECA cases were diagnosed (accounting for 3.2% of all cancer-related deaths) and 508,585 deaths were caused by ECA (accounting for 3.3% of all malignancies) (Bray et al., 2018). Surgical removal of tumors is the conventional treatment for most types of ECA. Although advances in surgical procedures, chemoradiotherapy, and immunotherapy have helped improve the overall survival (OS) of patients, the current 5-year survival rates of ECA patients still range from 15 to 25% (Pennathur et al., 2013). Therefore, in-depth exploration of potential prognostic and predictive markers, therapeutic targets, and mechanisms of ECA is a key imperative to improve the treatment outcomes and prognosis of ECA patients.

Due to the rapid advances in microarray and RNA-sequencing (RNA-seq) technology, multiple circular RNAs (circRNAs), microRNAs (miRNAs), and messenger RNAs (mRNAs) have been identified as important genes in ECA (Ritchie et al., 2015). CircRNAs are endogenous non-coding RNAs with closed-loop structures without 5′caps and 3′ tails (Kristensen et al., 2019). Recent studies have found that the mRNAs targeted by miRNAs in the circRNA–miRNA–mRNA–competing endogenous RNA (ceRNA) regulatory network could serve as a key therapeutic target in cancer. In addition, circRNAs were shown to play a vital role in the occurrence and development of cancers by positively or negatively regulating the circRNA–miRNA–mRNA–ceRNA axis or by acting as protein “sponges” in cancer cells. CircRNAs in the ceRNA network may indirectly regulate those target mRNAs by serving as miRNA “sponges” (Zhang et al., 2017; Wu et al., 2019). In a study, low expression of large tumor suppressor kinase 1 (*LATS1*) was found to be related to the tumor stage and poor prognosis of gastric cancer patients; in addition, circ*LARP4* was found to inhibit the malignant biological behavior of gastric cancer as a tumor suppressor *via* the modulation of the circ*LARP4*/miR-424-5p/*LATS1* axis (Yang et al., 2018).

Genome-wide analyses of ECA have indicated complex mutation situations and discovered significant gene mutations (including *TP53, MLL2*, *NOTCH1, and PTEN*), repeated copy number amplifications in *SOX2, TERT, and FGFR1*, as well as frequent deletion of RB1 (Chang et al., 2017; (Cancer Genome Atlas Research Network, 2017). However, previous ECA genome studies had some limitations, including relatively small sample size, low-coverage whole-genome sequencing, and one-sided analysis based only on chip data or sequencing data. Therefore, more comprehensive studies are required to overcome the shortcomings of previous studies.

The purpose of our study was to explore the potential prognostic and predictive markers and to unravel the mechanisms of ECA from our RNA-seq data, the Gene Expression Omnibus (GEO), and The Cancer Genome Atlas (TCGA) database. Differentially expressed circRNAs (DEcircRNAs), differentially expressed miRNAs (DEmiRNAs), and differentially expressed mRNAs (DEmRNAs) were identified using the Limma R package, and the circRNA–miRNA–mRNA–ceRNA regulatory network was constructed based on these differentially expressed genes. In addition, the enriched pathways were investigated by performing multiple functional and pathway enrichment analyses. Furthermore, the key target mRNAs in the ceRNA regulatory network were determined using survival Cox regression analyses. Finally, analyses of immune infiltration autophagy genes related to the key targets were performed. Our study may provide novel insights into the prognosis and treatment of ECA based on the pathogenetic mechanism.

## Methods

### Sample Collection

Between July and December 2020, a total of 20 pairs of tumor and adjacent tissues were collected from patients with pathologically confirmed ECA at the Department of Thoracic Surgery, Fujian Cancer Hospital. The Ethics Committee of the Fujian Cancer Hospital approved the use of human tissues (Project Ethics Number: SQ 2020-063-01), and informed consent was acquired. The study protocol conformed to the principles enshrined in the Declaration of Helsinki.

### RNA Sequencing

Six pairs of esophageal cancer and adjacent normal tissues from Fujian Cancer Hospital between July 1st and July 17th, 2020 were used for RNA-sequencing. First, we characterized circRNA transcripts by sequencing analysis of ribosomal RNA and linear RNA. The total RNA was extracted by the Trizol method. Then, every sample was sequenced on Illumina HiSeq yielding an average of 42.38 million reads, which were mapped to the human reference genome (GRCh38/hg38) by TopHat2 (v2.1.1). The CIRC explorer program (v2.2.3) was used with the fusion junctions obtained from TopHat2 to identify both the circularizing junction and the spliced sequence of circRNAs. The whole step of library construction and sequencing was performed at Shanghai Life genes Technology Co. Ltd.

### Datasets

The chip data of circRNA expression profiles, that is, GSE131969 (GPL 19978) were downloaded from the GEO (Barrett et al., 2013). The miRNA and mRNA expression and clinical information in TCGA were obtained from the University of California Santa Cruz (USCS) Xena. The circRNA microarray data included three ECA tissues and three adjacent normal esophageal tissues. The miRNA sequencing data included 185 ECA samples and 13 adjacent samples, and the mRNA sequencing data included 162 ECA samples and 11 adjacent samples.

### Differential Expression Analyses

The Limma R package (version 4.0.2) (Ritchie et al., 2015) and the edge R package (version 3.14.0) (Robinson et al., 2010) were used for differential analyses. The expressions of all differentially expressed genes (DEGs) were visualized by volcano maps and two-way clustering heat maps. Principal component analysis (PCA) was conducted with the “pca3d” R package to explore gene expression patterns of ECA and normal groups.

### Construction of the ceRNA and circRNA-RBP Network

MiRanda was used to predict the target miRNAs of the DEcircRNAs. Target mRNAs of DEmiRNAs were predicted using miRWalk (version 2.0) (Dweep, Gretz, 2015). The databases used for targeted mRNA prediction included miRWalk, Microt4, miRanda, mirbridge, miRDB, miRMap, and Targetscan. Overlapping mRNAs in six or more databases were considered the target mRNAs. Moreover, the ceRNA network was constructed and visualized by Cytoscape (version 3.6.1) (Shannon et al., 2003). The potential RNA binding proteins (RBPs) were analyzed by the CatRAPID database. A star rating score > 2 was considered indicative of strong binding effect. Cytoscape software was used to construct the circRNA–RBP binding network.

### Functional and Pathway Enrichment Analyses

The mRNAs in the ceRNA network were analyzed using Search Tool for the Retrieval of Interacting Genes (STRING) (Szklarczyk et al., 2019), Database for Annotation, Visualization, and Integrated Discovery (DAVID) (Dennis et al., 2003), and Metascape (Zhou et al., 2019). Metascape, DAVID, and STRING databases were used to conduct analyses based on DisGeNET (version 7.0) (Piñero et al., 2020), Gene Ontology (GO) (Ashburner et al., 2000), biological processes (BPs), Transcriptional Regulatory Relationships Unraveled by Sentence-based Text mining (TRRUST) database (version 2) (Han et al., 2018), and Kyoto Encyclopedia of Genes and Genomes (KEGG) (Kanehisa et al., 2017) analyses.

### Gene Set Variation Analysis

The enrichment scores of pathways were calculated using the GSVA (Hänzelmann et al., 2013), and a scoring matrix was obtained. Based on the matrix, the difference analysis was performed using Limma and visualized with a two-way clustering heat map.

### Survival Analysis

Based on the optimal cutoff calculated by the survminer R package (Alboukadel et al., 2020), the patients were divided into low and high gene expression groups. Between-group differences were evaluated using the log-rank test with the survival (version 3.2-7) R package (Therneau, 2020), and target genes that showed significant association with survival were chosen (*p* < 0.05). Furthermore, single-factor and multi-factor Cox regression analyses were performed on mRNAs with significant results. Finally, LIM domain containing 2 (*LIMD2*) with significant results was selected as a key target.

### Immune Infiltration Related Analysis

Based on TCGA database, CIBERSORT (Newman et al., 2015) was used to perform immune infiltration–related analysis, and immune cells were visualized between groups. Based on the ratio of various immune cells, Spearman’s correlation analysis was performed to assess the correlation between immune cells and between targets and immune cells.

### Autophagy Correlation Analysis

The autophagy-related genes were obtained from the Human Autophagy Database (HADb). The expressions of key target *LIMD2* and autophagy-related genes were analyzed by Spearman’s correlation analysis, and the *p-*values were adjusted using the BH method.

### Gene Set Enrichment Analysis

GSEA (Reimand et al., 2019), performed by R package cluster Profiler (version 3.8.0) (Yu et al., 2012), was used to analyze the significant function and pathway difference between high- and low- *LIMD2* groups. *P-*value <0.05 and FDR *q-*value < 0.25 were set as the cutoff criteria.

### Single-Factor and Multi-Factor Cox Regression Analyses

Among the clinical variables, age, M stage, N stage, T stage, sex, tumor stage, and *LIMD2* expression were selected for univariate Cox regression analysis. Multivariate Cox regression analyses were conducted using variables that showed significant results in univariate Cox regression analysis.

### qRT-PCR Validation for the Expression of *LIMD2*

Quantitative reverse transcription-polymerase chain reaction (qRT-PCR) was applied to verify the expression of the target LIMD2 in 20 pairs of ECA and adjacent normal esophageal tissues. The primers of LIMD2 were purchased from BioSune (Shanghai, China) (Table 2). An RT2162; All-in-One Mix with dsDNase (Monad Biotech Co. Ltd., Shanghai, China) was used to synthesize cDNA from 1 µg of total RNA. The qRT-PCR analyses were conducted on the Quant Studio 6 Flex qRT-PCR system (Applied Biosystems, Thermo Fisher Scientific Co. Ltd., United States) using the Hieff ®qPCR SYBR® Green Master Mix, Low Rox (Yeasen, Biotechnology Co., Ltd, Shanghai, China). The reaction was: 95°C for 10 min, then 40 cycles of 95°C for 15 s and 6°C for 1 min. The reference gene was GAPDH, and the relative gene expression levels were calculated using the 2−ΔΔCt method.

### Statistical Analysis

Statistical analyses were performed using R software (version 3.6.1), GraphPad Prism (version 8.0.1.244), SPSS (version 24.0), and the bioinformatics tools mentioned above. Differential expressions of genes were obtained by two-tailed Student’s t-test. The Benjamini and Hochberg FDR method was conducted to adjust the *p-*values. Enrichment analyses were analyzed using the hypergeometric test and Bonferroni correction. The outcomes were expressed as the mean ± SD and between-group differences were assessed using the paired Student’s *t*-test and Wilcoxon rank sum test. *P* values < 0.05 were considered indicative of statistical significance.

## Results

### Data Collection and Preprocessing

**FIGURE 1:**
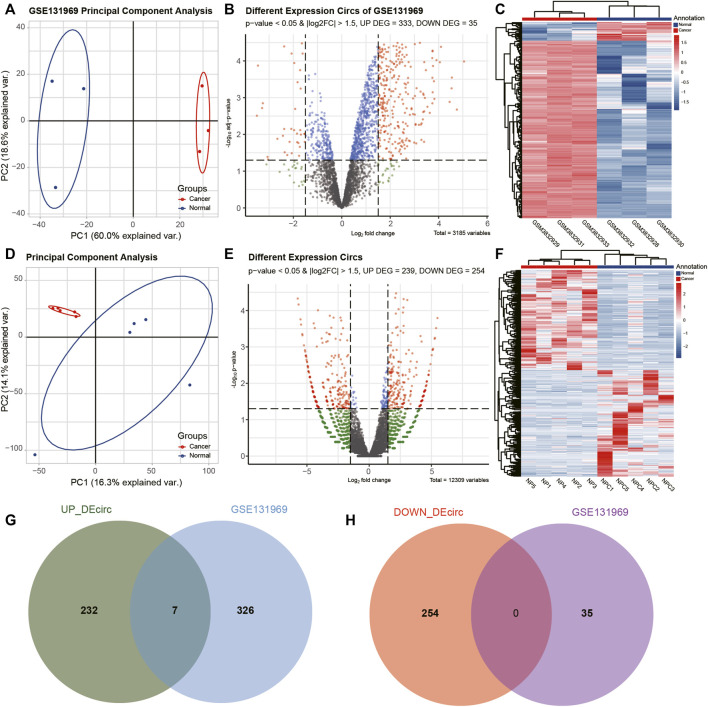
Identification of differentially expressed circRNAs (DEcircRNAs) in esophageal cancer (ECA). **(A, D)** Principal component analysis (PCA) of circRNA expression between ECA and normal groups in GSE131969 and the present RNA-sequencing (RNA-seq) data. **(B, E)** Volcano plot of the distributions of all DEcircRNAs in GSE131969 and the current RNA-seq data. **(C, F)** Hierarchical clustering heatmap of dysregulated circRNAs between ECA and the adjacent normal tissues in GSE131969 and the present RNA-seq data. **(G, H)** Comparison of differentially expressed circRNAs identified in GSE131969 and our RNA-seq data.

**FIGURE 2:**
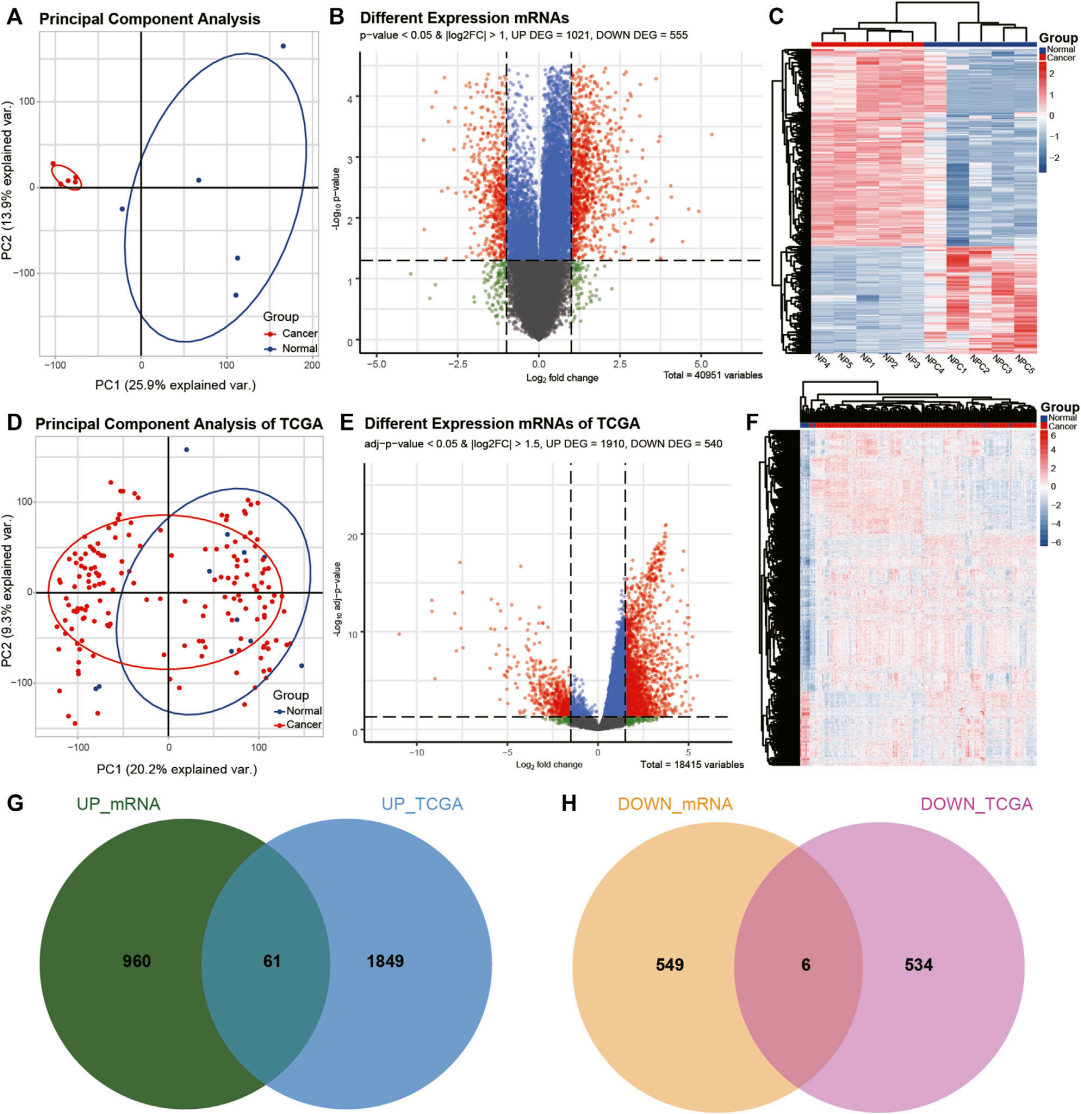
Identification of differentially expressed mRNAs (DEmRNAs) in ECA. **(A, D)** PCA of mRNA expression between ECA and normal groups in TCGA cohort and the present RNA-seq data. **(B, E)** Volcano plot of the distributions of all differentially expressed mRNAs in TCGA cohort and the current RNA-seq data. **(C, F)** Hierarchical clustering heatmap of dysregulated mRNAs between ECA and the adjacent normal tissues in TCGA cohort and the present RNA-seq data. **(G, H)** Comparison of differentially expressed mRNAs identified in TCGA cohort and our RNA-seq data.

**FIGURE 3:**
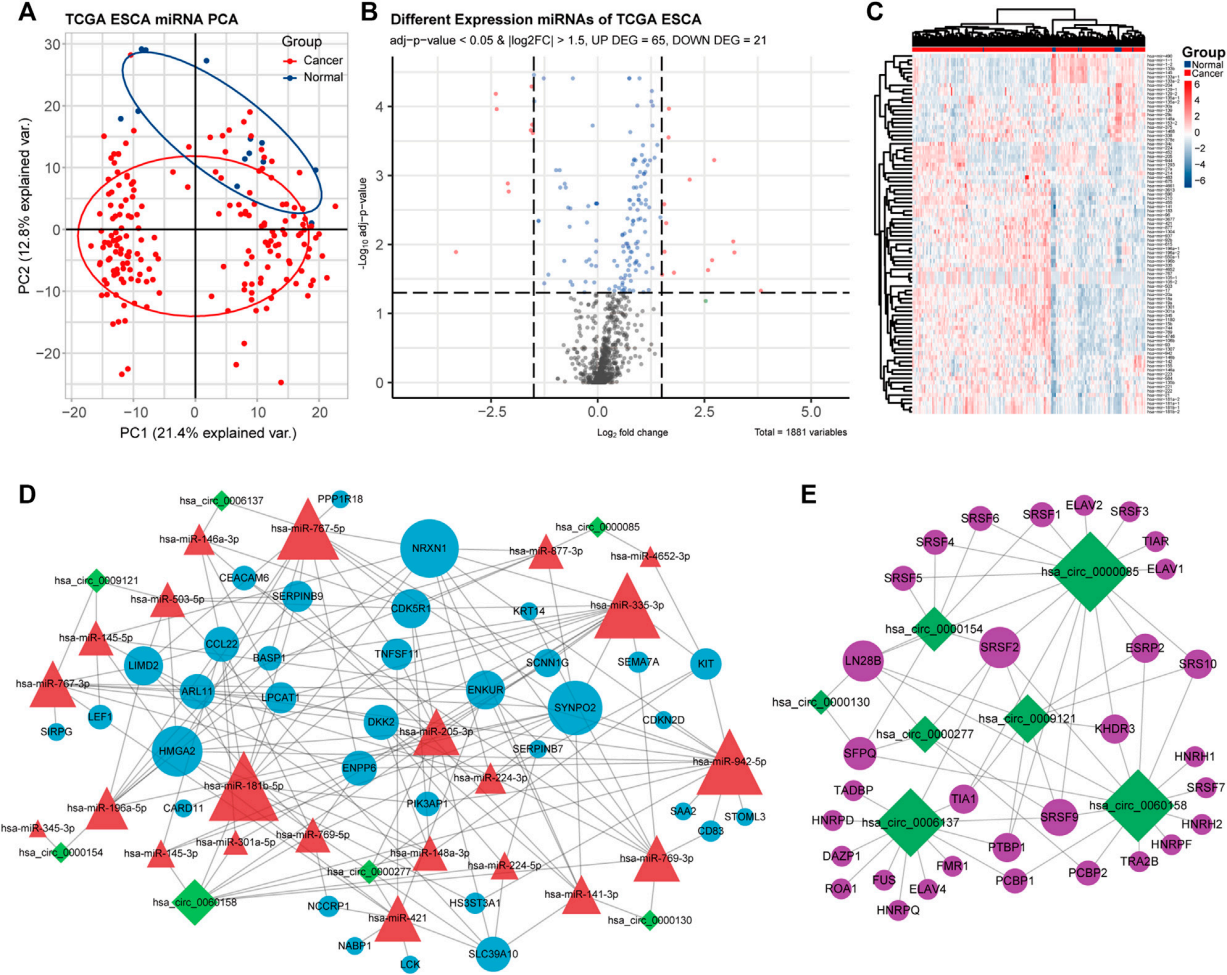
Identification of differentially expressed miRNAs (DEmiRNAs) in ECA, the ceRNA network, and the circRNA–RNA binding protein (RBP) network. **(A)** PCA of miRNA expression between ECA and normal groups in TCGA cohort. **(B)** Volcano plot of the distributions of all DEmiRNAs in TCGA cohort. **(C)** Hierarchical clustering heatmap of dysregulated miRNAs between ECA and the adjacent normal tissues in TCGA cohort. **(D)** CeRNA network of circRNA–miRNA–mRNA in ECA; **(E)** circRNA–RBP binding network.

The differences between the clustering of the circRNAs (Figures 1A,D), mRNAs (Figures 2A,D), and miRNAs (Figure 3A) of the ECA and adjacent samples were analyzed. The results showed that most principal components could separate or tended to separate ECA samples from normal esophageal tissues.

### Identification of DEcircRNAs, DEmiRNAs, and DEmRNAs

CircRNAs in GSE131969 and RNA-seq data (*p-* value <0.05 and |log2 FC| > 1.5), mRNAs in RNA-seq data (*p-* value <0.05 and |log2 FC| > 1) and TCGA dataset (*p* value <0.05 and |log2 FC| > 1.5), as well as miRNAs inTCGA (*p* value <0.05 and |log2 FC| > 1.5) were analyzed. A total of 368 DEcircRNAs were identified in GSE131969 (Figure 1B), 493 DEcircRNAs were identified in RNA-seq data (Figure 1E), 1576 DEmRNAs were identified in RNA-seq data (Figure 2B), 2450 DEmRNAs were identified in TCGA (Figure 2E), and 86 DEmRNAs were identified in TCGA (Figure 3B). The DEcircRNAs in the GEO (Figure 1C) and RNA-seq data (Figure 1F) and DEmRNAs in RNA-seq data (Figure 2C) and TCGA (Figure 2F), as well as DEmiRNAs in TCGA (Figure 3C), were visualized using the hierarchical cluster maps. Seven upregulated circRNAs (Figure 1G), zero downregulated circRNAs (Figure 1H) and 61 upregulated mRNAs (Figure 2G) and six downregulated mRNAs (Figure 2H) were significantly differentially expressed in both datasets. Finally, seven DEcircRNAs in the GSE131969 (Supplementary Figure S1A) and RNA-seq data (Supplementary Figure S1B) were visualized.

### Construction of the CeRNA Network and CircRNA–RBP Network

**FIGURE 4:**
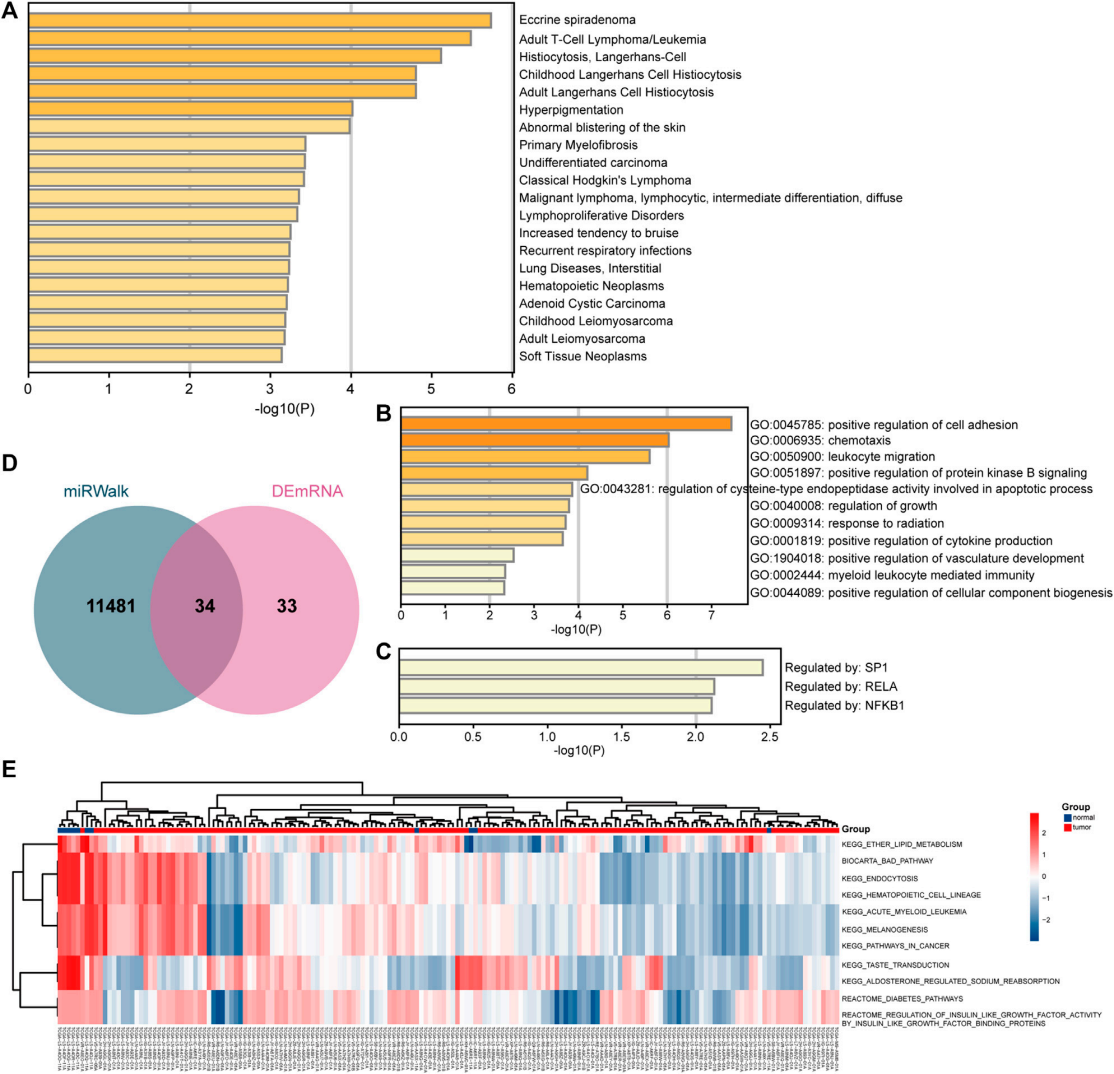
Biological enrichment analyses of target mRNAs. **(A)** Enrichment results based on the DisGeNET database. **(B)** Enrichment results based on the gene ontology (GO) and biological process (BP) databases. **(C)** Enrichment results based on the TRRUST database. **(D)** Comparison of target mRNAs predicted by miRNAs and differentially expressed mRNAs. **(E)** GSVA analysis of key targets.

The miRNAs targeted by the seven DEcircRNAs were predicted and 22 circRNA–miRNA pairs were identified. The mRNAs targeting the miRNAs were identified, and 34 mRNAs were obtained through the intersection with DEmRNAs (Figure 4D). Subsequently, the differential expression of the 34 mRNAs TCGA database (Supplementary Figure S3A) and RNA-seq data were visualized (Supplementary Figure S3B). A ceRNA network was established based on seven circRNAs, 22 miRNAs, and 34 mRNAs (Figure 3D). The circRNA–RBP network was constructed, including seven circRNAs and 37 RBPs (Figure 3E).

### Functional and Pathway Enrichment Analyses

The enriched terms analyzed based on the DisGeNET database are shown in Figure 4A, including adult T-Cell lymphoma and childhood Langerhans cell histiocytosis. The enriched GO terms are shown in Figure 4B, including positive regulation of cell adhesion and positive regulation of protein kinase B signaling. The enriched terms analyzed based on the TRRUST database were SP1, RELA, and NFKB1 (Figure 4C). Moreover, GSVA showed enrichment of ether lipid metabolism or acute myeloid leukemia in the cancer group (Figure 4E). Based on the DAVID database, the outcomes showed that mRNAs were enriched in positive regulation of T cell activation and cell migration (BP), extracellular space, plasma membrane (cellular components, CCs), DNA binding (Molecular Functions, MFs), and NF-kappa B signaling pathway (KEGG, Supplementary Figure S2A). Based on the STRING database, the results showed that targets were enriched in positive regulation of lymphocyte activation and immune system process (BPs), extracellular space (CC), protein binding and DNA binding (MFs), NF-kappa B pathway and ether lipid metabolism (KEGG), and regulation of PI3K/AKT signaling (record control the memory, RCTM, Supplementary Figure S2B).

### Survival Analysis

**FIGURE 5:**
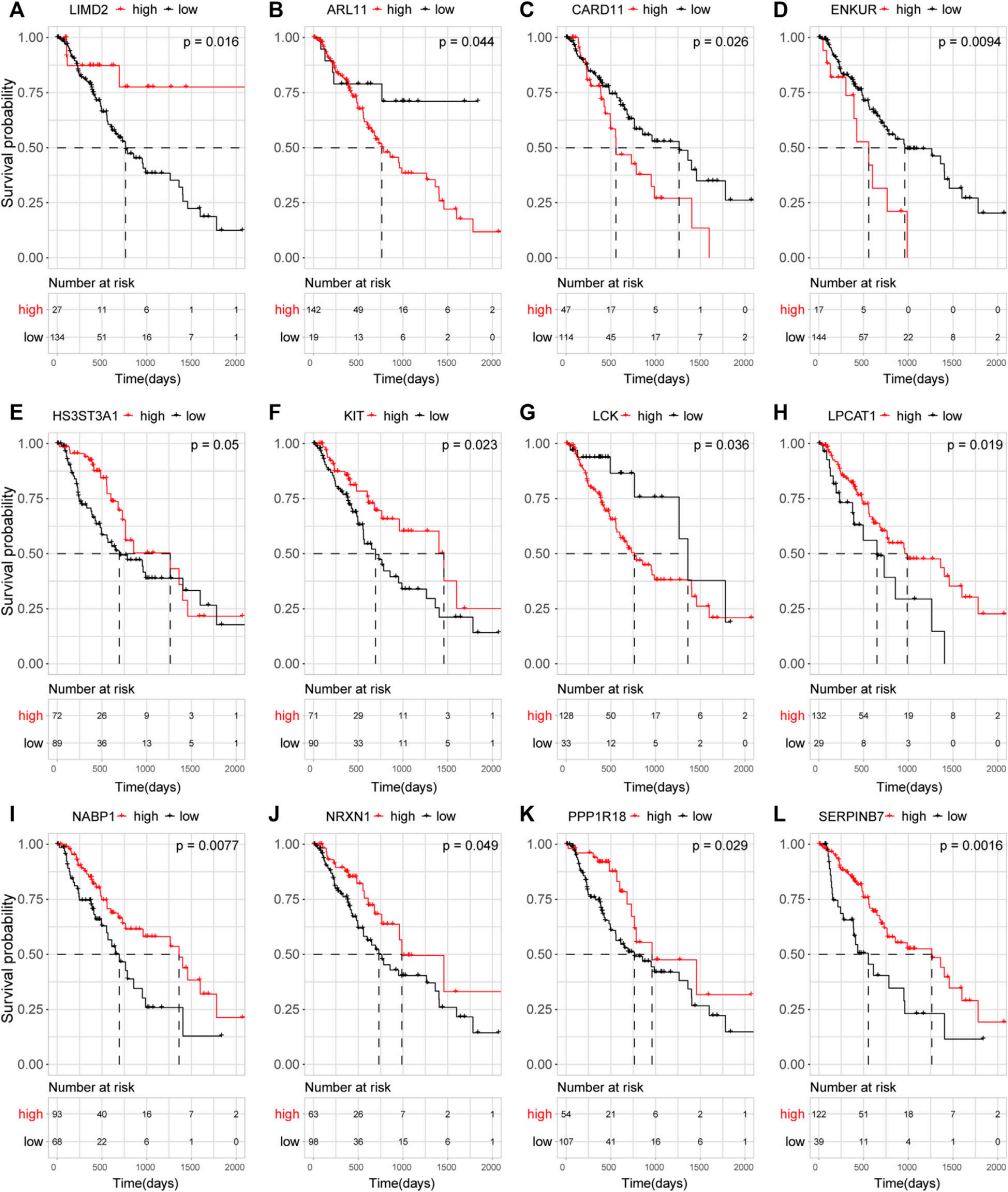
Effect of target mRNA expression on the OS of ECA patients in TCGA database. Kaplan–Meier (KM) curves demonstrating the distinct outcomes of ECA patients. **(A–L)** KM curve of *LIMD2* **(A)**, *ARL11* **(B)**, *CARD11* **(C)**, *ENKUR* **(D)**, *HS3ST3A1* **(E)**, *KIT* **(F)**, *LCK* **(G)**, *LPCAT1* **(H)**, *NABP1* **(I)**, *NRXN1* **(J)**, *PPP1R18* **(K)**, and *SERPINB7* **(L)**.

Targets that showed a significant association with survival (*p* < 0.05) included *LIMD2*, *ARL11, and SERPINB7* (Figures 5A–L). *LIMD2* with significant regression results was selected as a key target.

### Immune Infiltration Analysis

**FIGURE 6:**
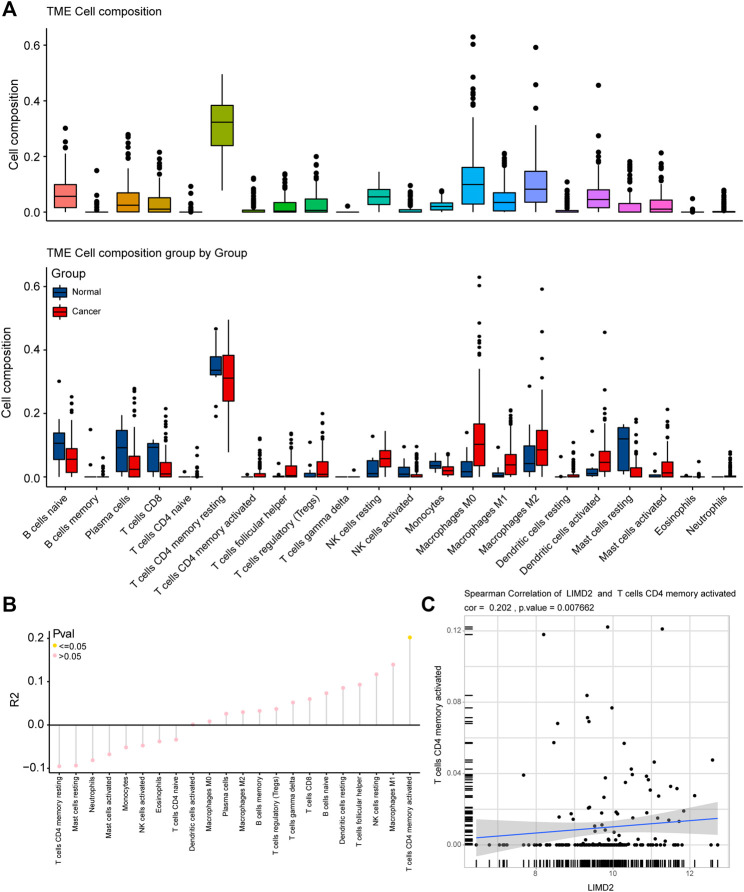
Immune infiltration correlation analysis in ECA based on TCGA cohort. **(A)** Fraction of the immune tumor microenvironment (TME) cells in the ECA and normal groups, and TME cells between the ECA and normal groups are analyzed in the figure given below. Within each group, the scattered dots represent the expression values of TME cells. The bottom and top of the boxes are the 25th and 75th percentiles (interquartile range). The whiskers encompass 1.5 times the interquartile range. **(B)** Immune infiltration correlation analysis; **(C)** Plots showing Spearman’s correlation of *LIMD2* and memory activated CD4 T cells.

The composition of immune cells in the tumor microenvironment (TME) in the ECA and normal groups was analyzed. We observed significant differences in TME cell infiltration and composition in the two groups, including memory activated CD4 T cells and M1 macrophages (Figure 6A). The *R* ^2^ of TME cells was also calculated, and the results showed that memory activated CD4 T cells had statistical significance (Figure 6B). A positive relationship was observed between *LIMD2* and memory activated CD4 T cells (Figure 6C). These analyses indicated a positive correlation of the ECA group with immune-relevant signatures.

### Analysis of *LIMD2* and Autophagy-Related Genes

**FIGURE 7:**
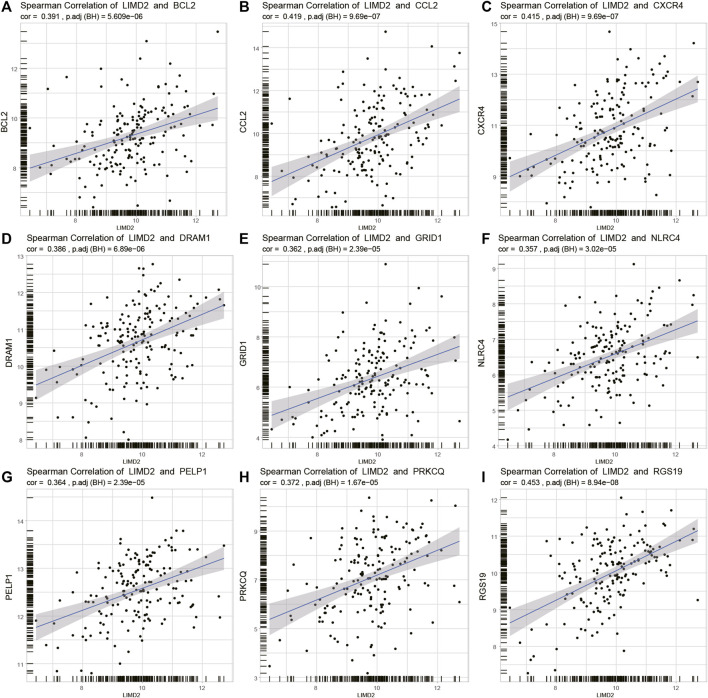
Plots showing Spearman’s correlation of *LIMD2* and autophagy-related genes in TCGA cohort. In every plot, the X-axis represents *LIMD2* and the Y-axis represents autophagy-related genes. **(A–I)** Spearman’s correlation of *LIMD2* and *BCL-2* **(A)**, *CCL2* **(B)**, *CXCR4* **(C)**, *DRAM1* **(D)**, *GRID1* **(E)**, *NLRC4* **(F)**, *PELP1* **(G)**, *PRKCQ* **(H)**, and *RGS19* **(I)**. Dashed line in each plot is the regression line.

Several studies have indicated the importance of autophagy in the development of ECA (Chen et al., 2020; Li et al., 2020). We investigated the relationship between *LIMD2* and autophagy-related genes (Figures 7A–I). The top nine autophagy-related genes that showed a positive relationship with *LIMD2* were identified, including *BCL-2*, *CCL2*, *RGS19, DRAM1*, *GRID1*, *NLRC4*, *PELP1*, *PRKCQ*, and *RGS19*.

### GSEA

The results demonstrated that the ECA patients with high expression of *LIMD2* showed enrichment of the following pathways: systemic lupus erythematosus, DNA replication, and T cell receptor signaling pathway (Supplementary Figures S4A–I).

### Cox Regression Analysis

Univariate and multivariate Cox regression analyses were performed to explore independent predictors of survival in ECA patients. A total of 162 ECA patients were included in the regression analysis. In univariate Cox regression analysis, *LIMD2*, M stage, N stage, and tumor stage showed a strong association with OS. Based on the results of univariate Cox analysis, multivariate Cox regression analysis was performed to analyze the effect of *LIMD2* and other related clinical phenotypes on the prognosis of ECA. In multivariate Cox regression analysis, *LIMD2* showed a significant correlation with OS (Table 1, Supplementary Figure S5). The results showed that *LIMD2* is an independent predictor of OS in ECA patients.

**TABLE 1:** Results of univariate analysis and multivariate analysis showing prognostic factors for OS.

Variables	Univariate analysis			Multivariate analysis		
	HR	95% CI	*p* Value	HR	95% CI	*p* Value
Age						
≥60 (77/137)	0.9911	0.574–1.711	0.974	—	—	—
Tumor_central location						
Mid (36/142)	8.6e-01	0.391–1.893	0.709	—	—	—
Proximal (6/142)	3.75e-08	0-Inf	0.997	—	—	—
M stage						
M1 (8/143)	5.32	2.449–11.576	2.45e-05	2.149	0.9242–4.997	0.0756
N stage						
N1-N3 (77/143)	3.504	1.82–6.744	0.000175	2.088	0.946–4.611	0.0685
T stage						
T3-T4 (80/143)	1.2498	0.719–2.173	0.43	—	—	—
Gender						
Male (121/143)	2.366	0.852–6.572	0.0984	—	—	—
Tumor stage						
Stage iii-stage iv (58/143)	3.398	1.9–6.076	3.68e-05	2.078	0.98–4.406	0.0564
Alcohol history						
Yes (97/140)	0.674	0.386–1.177	0.166	—	—	—
Smoking history						
2 (30/125)	1.65699	0.6496–4.226	0.2905	—	—	—
3 (28/125)	0.96495	0.3601–2.585	0.9434	—	—	—
4 (26/125)	2.38885	0.9319–6.123	0.0698	—	—	—
BMI						
>30 (15/134)	0.3895	0.122–1.244	0.1116	—	—	—
25-30 (72/134)	0.462	0.1757–1.216	0.1177	—	—	—
18.5-25 (39/134)	0.456	0.181–1.152	0.0968	—	—	—
*LIMD2*						
Low (94/143)	2.9065	1.413–5.981	0.00375	2.629	1.2486–5.537	0.0109

HR, hazard ratio; CI, confidence interval; Smoking history: 1, lifelong non-smoker (less than 100 cigarettes smoked in lifetime); 2, current smoker (includes daily smokers and non-daily smokers or occasional smokers); 3, current reformed smoker for more than15 years (greater than 15 years); 4, current reformed smoker for ≤15 years (less than or equal to 15 years).

### Validation of *LIMD2* by QRT-PCR

**FIGURE 8:**
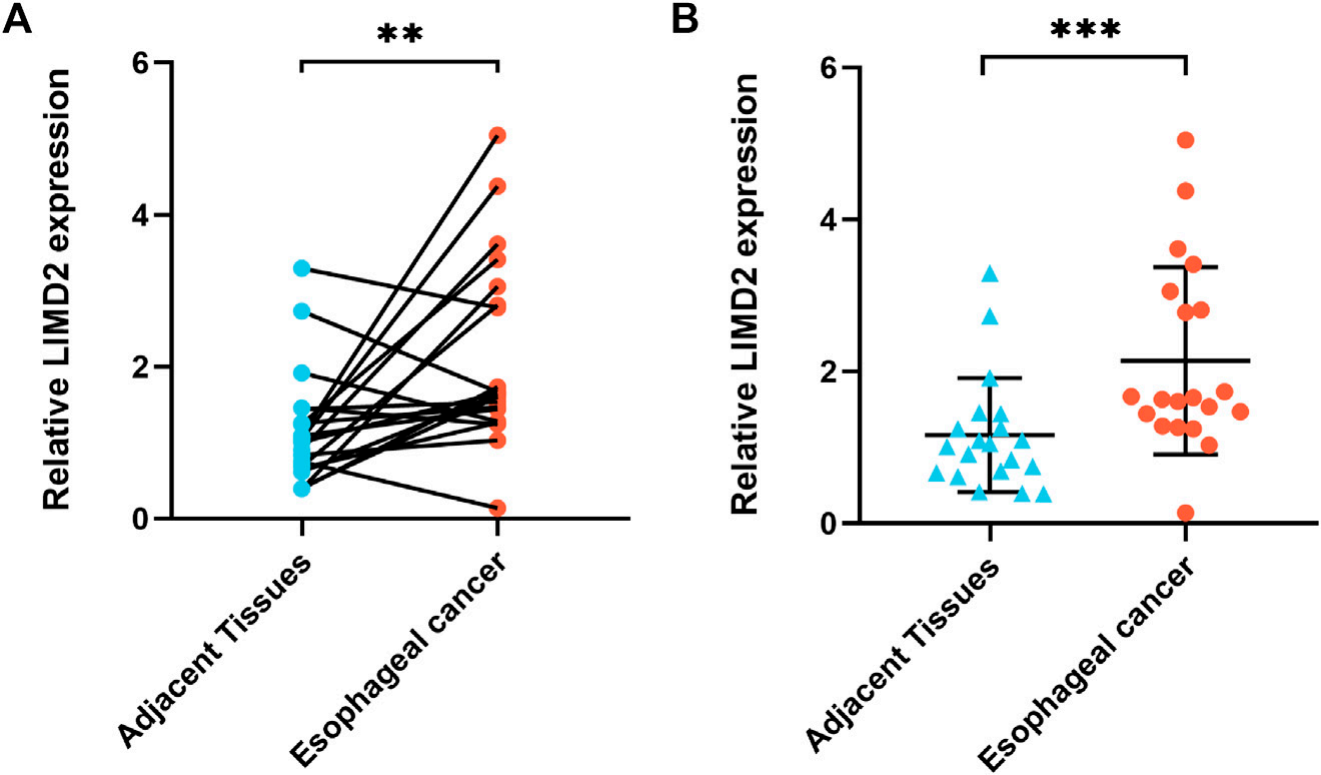
Validation of the expression of target *LIMD2*. The *LIMD2* expression in ECA (*n* = 20) and adjacent normal tissues (*n* = 20) was evaluated by qRT-PCR; the results were analyzed using the paired sample *t-*test **(A)** and Wilcoxon rank sum test **(B)**. Results expressed as mean ± standard deviation (SD). *p < 0.01 and **p < 0.001.

To verify the RNA-seq and bioinformatics results, the target *LIMD2* was chosen for validation by quantitative reverse-transcription polymerase chain reaction (qRT-PCR) with primers (Table 2). The results showed significantly higher expression of *LIMD2* in ECA tissues compared with that of normal tissues (Figure 8).

**TABLE 2:** Primers for *LIMD2* and *GAPDH*.

Primers	Forward primers (5′to 3′)	Reverse primers (5′to 3′)
qPCR-*LIMD2*	TTT​TCC​ACA​ACT​CTT​GCT​TCT​GC	AAC​CCC​TCG​TCG​TAG​TTG​CCT
qPCR-*GAPDH*	GGA​GCG​AGA​TCC​CTC​CAA​AAT	GGC​TGT​TGT​CAT​ACT​TCT​CAT​GG

## Discussion

In this study, seven circRNAs, 22 miRNAs, and 34 target mRNAs in the circRNA–miRNA–mRNA–ceRNA regulatory network were identified as crucial genes in ECA; in addition, the nuclear factor-k-gene binding (NF-κB) and phosphatidylinositol-3 kinase/protein kinase B (PI3K-Akt) signaling were identified as the most enriched pathways. Survival Cox regression analyses indicated that the target *LIMD2* in the ceRNA network may act as an independent predictor of OS in ECA patients. On further analyses of immune infiltration and autophagy genes related to target *LIMD2*, *LIMD2* showed a close linkage with immunity and autophagy in ECA. Furthermore, we verified the high expression level of *LIMD2* by qRT-PCR based on 20 pairs of ECA and normal samples. Hence, *LIMD2* is a potential molecular marker of prognostic and predictive significance in ECA.

The ceRNA networks may play a vital role in the development of cancer (Liu et al., 2021). Seven circRNAs, 22 miRNAs, and 34 mRNAs were identified in the ceRNA network. The increase of has_circ_0000154 (circDCAF6) identified in our study was related to tumor invasion, positive lymph node metastasis, and a higher TNM stage in GC patients. It could serve as an independent prognostic indicator (Wu et al., 2019). A total of 12 identified mRNAs (*HMGA2, CCL22, CDKN2D, CEACAM6, DKK2, KIT, KRT14, LEF1, LPCAT1, NCCRP1, NRXN1, and PPP1R18*) were found to be related to ECA. In a previous study, high-mobility group AT-hook 2 (*HMGA2*) was shown to regulate transcription by inducing structural alterations in the chromatin (Mansoori et al., 2021). Several studies have shown that *HMGA2* is re-expressed in most tumors and plays a vital role in tumorigenesis (Lu et al., 2021). The stability of *HMGA2* may be regulated by hepatitis B X-interacting protein (*HBXIP*) *via* the Akt-PCAF pathway, thereby promoting the growth of ECA cells (Wu et al., 2020). All 22 DEmiRNAs in the ceRNA network have been reported to be related to cancer. MiR-141-3p plays an important role in various carcinomas (Huang et al., 2017), and it was found to be highly upregulated in ECA cells (Phatak et al., 2021). A recent study reported that miR-141-3p may inhibit the expression of pleckstrin homology domain leucine-rich repeat protein phosphatase-2 (*PHLPP2*), a negative regulator of the PI3K/AKT signaling, and could serve as an biomarker in ECA (Ishibashi et al., 2018).

CircRNAs with RBP binding sites can act as sponges for RBPs and may indirectly modulate their functions (Zhang et al., 2017). In this study, the six identified RBPs (*SRSF1, SRSF2, PCBP2, TIA1, FUS, and FMR1*) were closely related to ECA. *PCBP2* performs multiple functions, such as stabilization of mRNAs and silencing or promotion of translation (Ye et al., 2016). Several studies have shown that *PCBP2* may promote tumor growth. Ye et al. found that *PCBP2* regulates the proliferation and apoptosis of ESCC cells and may serve as a novel therapeutic target in ESCC (Ye et al., 2016). Our findings are consistent with those of the previous study.

In our study, multiple diseases were enriched in ECA, including adult T cell lymphoma (ATL), childhood Langerhans cell histiocytosis, and classical Hodgkin’s lymphoma that are strongly associated with immune cells. ATL is a T cell lymphoproliferative tumor of mature CD4^+^ CD25 ^+^ T cells (Mehta-Shah et al., 2017), and Langerhans cell histiocytosis is characterized by the accumulation of Langerhans cells and antigen-presenting cells (Rayamajhi et al., 2020). Our results also revealed a close relation of many enriched GO and KEGG terms with immune response, including positive regulation of T cell activation and immune system process. GSEA showed that immunity was strongly associated with ECA, including systemic lupus erythematosus and the T cell receptor signaling pathway. The NF-κB signaling and PI3K/AKT signaling pathways were the most enriched pathways. Zheng et al. depicted the entire immune landscape, including the innate and acquired immune cell map, in ESCC and adjacent tissues; their work revealed that ESCC is enriched in immune-suppressive cell mass (Zheng et al., 2020). Tong et al. revealed that 14-3-3ζ may enhance the invasion and growth of ESCC cells by inhibiting the *S1PR2* protein expression *via* the NF-κB pathway (Tong et al., 2018). GSVA indicated enrichment of ether lipid metabolism in ECA. Disorders of ether lipid metabolism are vital signs of tumors, which serve as the basis of tumor pathogenicity (Piano et al., 2015). Cao et al. found that lncRNAs may interact with their adjacent coding RNAs to modulate ether lipid metabolism (Cao et al., 2013).

Furthermore, we found that 12 genes were strongly related to OS. We included these 12 mRNAs in Cox regression analyses and identified *LIMD2* as a vital prognostic factor. In our study, high expression of *LIMD2* was associated with better OS in ECA. However, Zhang et al. found that high expression of *LIMD2* may enhance the progression of non–small cell lung carcinoma (NSCLC); in addition, the overexpression of *LIMD2* was closely related to lymph node metastasis, distant metastasis, and advanced stage. The survival time of NSCLC patients with the overexpression of *LIMD2* was shorter than that of patients with lower expression, indicating that *LIMD2* may serve as a therapeutic target in NSCLC (Zhang et al., 2019). Our study indicated that *LIMD2* may serve as an independent predictor of prognosis in ECA patients. In the TME cell composition analysis, Yu et al. found that the high-immunity group of cutaneous melanoma specimens had the highest level of memory activated CD4 T cells, and the OS rate was poor (Yu et al., 2020). A recent research unraveled the connection between clinical information and immune signatures in GC. They noticed that the high-risk group showed greater proportion of memory activated CD4 T cells and M1 macrophages (Liu et al., 2020). Our study has also demonstrated positive relationship of *LIMD2* with nine autophagy-related genes. In our study, *BCL-2*, an autophagy-related gene showed a positive relationship with *LIMD2*, and *BCL-2*–associated athanogene 3 (*BAG3*) was shown to be involved in multiple biological processes, including cell proliferation, cell vitality, and apoptosis (Rosati et al., 2011). Compared with Barrett’s metaplasia (BE) and normal samples, the amount of T lymphocytes with downregulation of *BCL-2* was notably increased in EAC (Berndt et al., 2010). In short, *LIMD2* is a potential marker of prognostic and predictive significance in ECA.

Nevertheless, some limitations of our study should be considered while interpreting the results. First, owing to the use of several datasets in our analyses, the effect of inter-batch differences on our results cannot be ruled out. In addition, further studies with a larger sample size are required to verify the high expression of *LIMD2* in ECA. Moreover, the sample size of squamous carcinoma and adenocarcinoma was too small to be analyzed separately. To avoid unreliable results, we analyzed both histological subtypes. We will further expand the sample size to explore the differences between different subtypes. Finally, further *in vitro* and *in vivo* biological experiments on immunity and autophagy are required for in-depth characterization of the functions of the identified targets and potential mechanisms in ECA.

In conclusion, we identified seven circRNAs, 22 miRNAs, and 34 mRNAs in the ceRNA network in ECA. The NF-κB and PI3K/Akt signaling pathways were the most enriched pathways in ECA. Furthermore, *LIMD2* was notably upregulated in ECA tissue samples and may serve as a potential prognostic and predictive marker that is closely associated with immunity and autophagy. Our study provides insights into the pathogenesis, prognosis, and therapeutic strategy for ECA.

## Data Availability

The datasets generated and analyzed for this study can be found in the National Center for Biotechnology Information Gene Expression Omnibus (Barrett et al., 2013) repository under the accession number GSE131969.
