# Supplementary material for: LIMD2 is a Prognostic and Predictive Marker in Patients With Esophageal Cancer Based on a ceRNA Network Analysis
Source: Front Genet. 2021 Nov 18;12:774432. doi: 10.3389/fgene.2021.774432 (PMC8636797; doi:10.3389/fgene.2021.774432)
Supplement: Supplementary file 1 [file DataSheet1.pdf]

Supplementary Material

1 Supplementary Figures

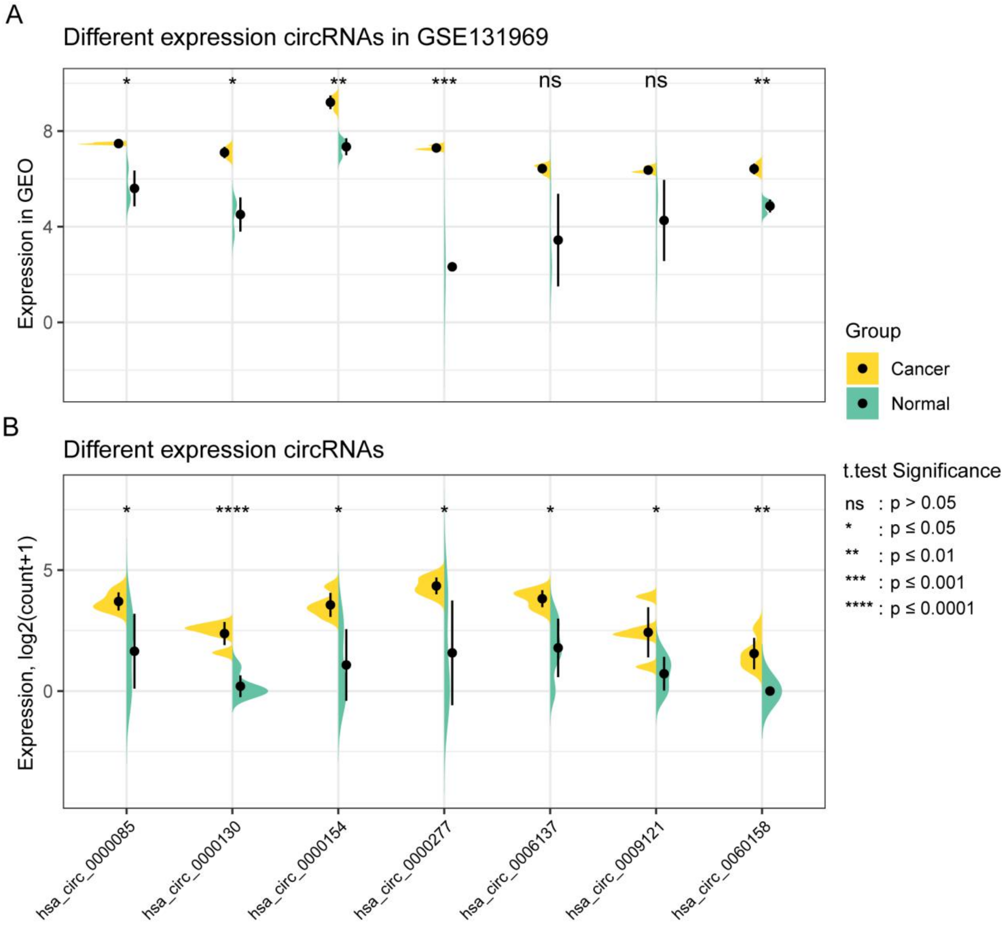

Supplementary Figure 1. Expressions of seven differentially expressed circRNAs in GSE131969 and our RNA-seq data, respectively. **(A, B)** Differentially expressed circRNAs between ECA and the adjacent normal tissues in GSE131969 and the present RNA-seq data.

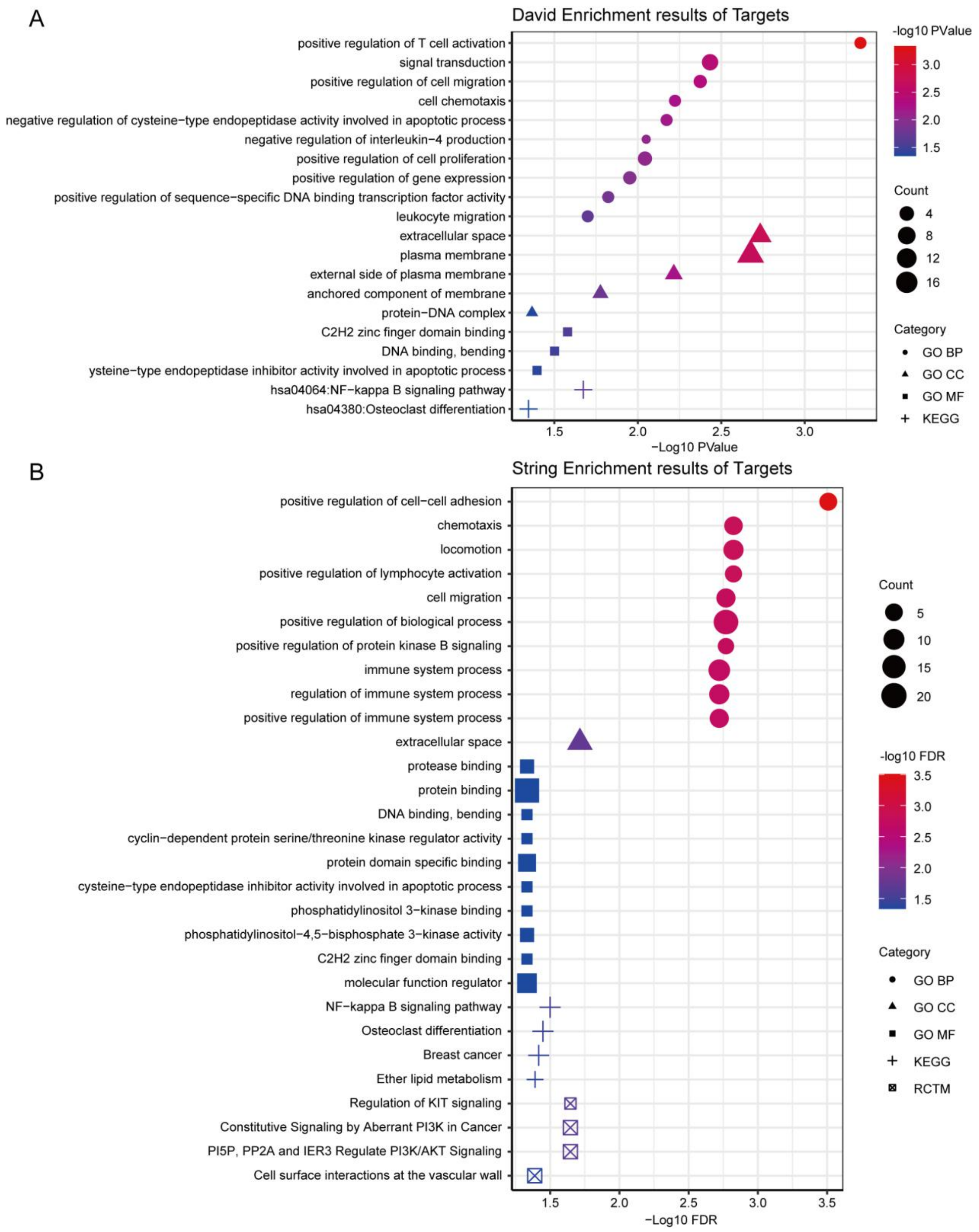

Supplementary Figure 2. Functional annotation of target mRNAs. **(A)** Functional enrichment of targets using the David database; **(B)** functional enrichment of targets using the String database.

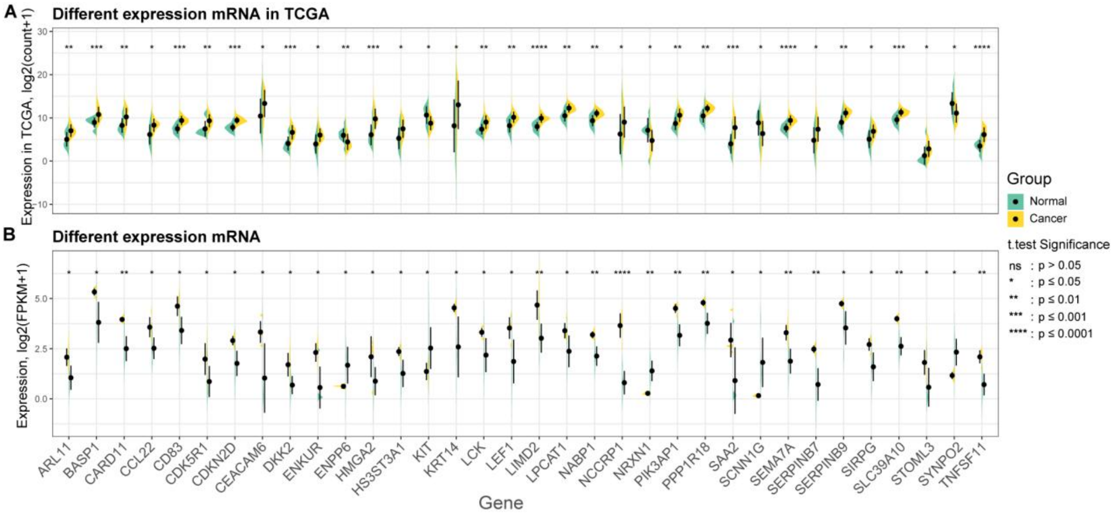

Supplementary Figure 3. Differential expression of target mRNAs in the TCGA database and our RNA-Seq data. **(A)** Differential expression of mRNAs in the TCGA database; **(B)** differential expression of mRNAs in our RNA-Seq data.

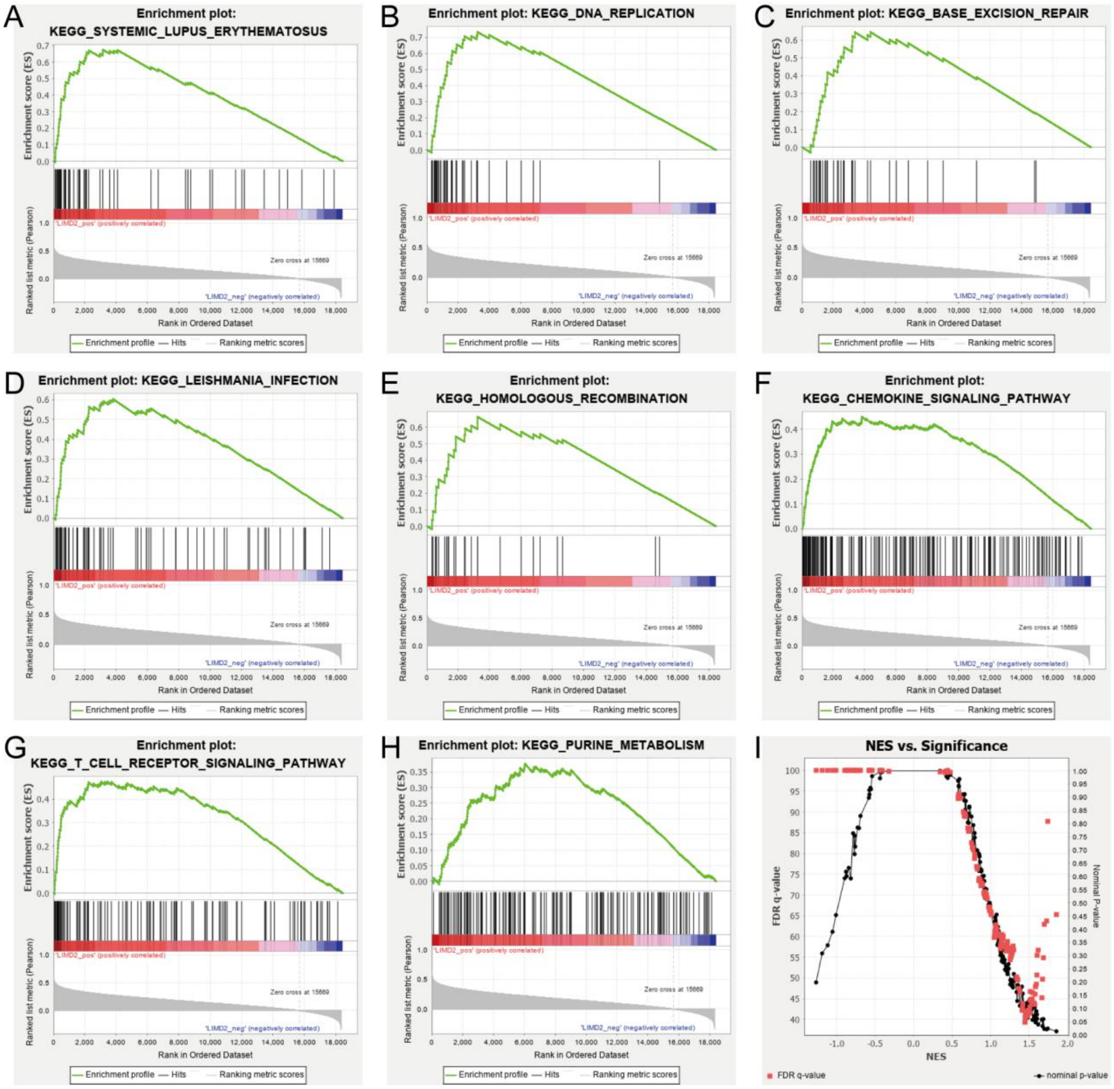

Supplementary Figure 4. Gene set enrichment analysis (GSEA) of key target mRNAs in ECA. **(A-H)** Gene set enrichment analysis revealed that systemic lupus erythematosus **(A)**, DNA replication **(B)**, base excision repair **(C)**, leishmania infection **(D)**, homologous recombination **(E)**, chemokine signaling pathway **(F)**, T cell receptor signaling pathway **(G)**, and purine metabolism **(H)** were enriched in ECA. **(I)** Normalized enrichment score (NES) and corresponding significance. P < 0.05.

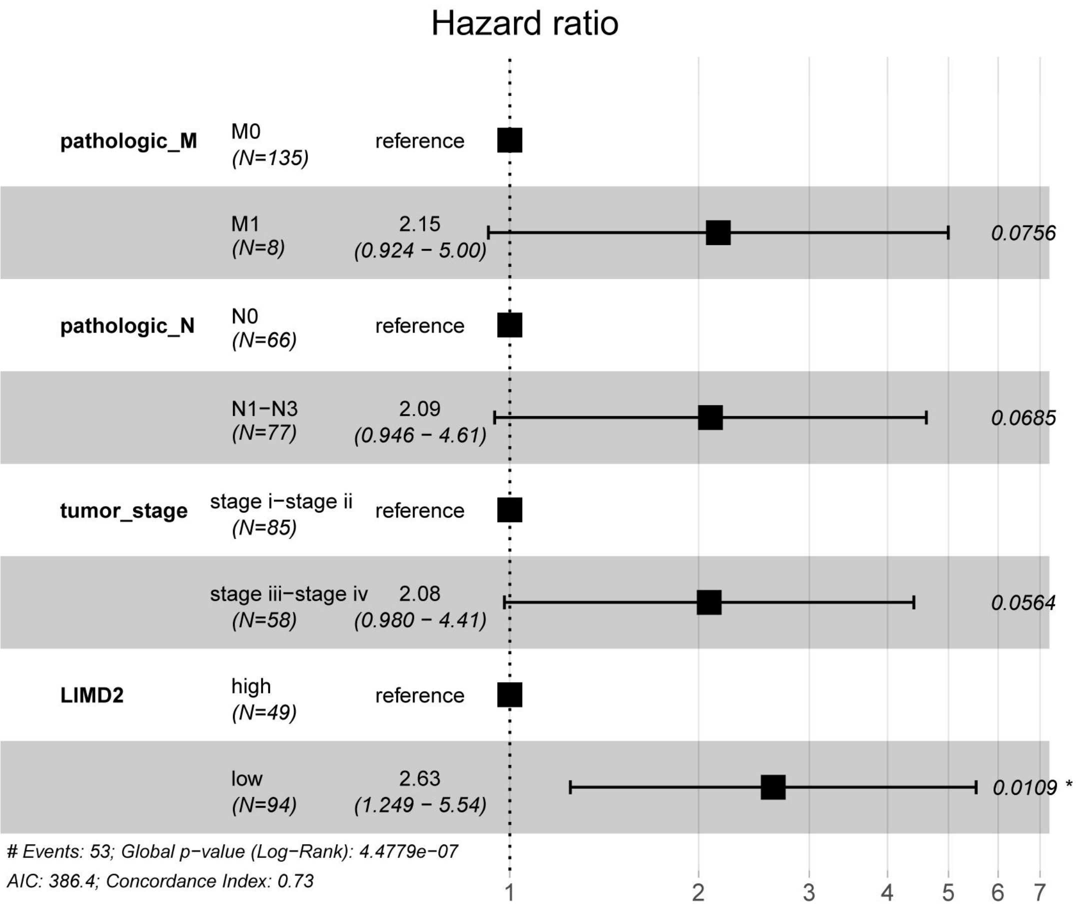

Supplementary Figure 5. Relation between *LIMD2* expression in ECA and clinical variables based on TCGA cohort. Cox regression analysis was performed to identify factors associated with *LIMD2* in ECA patients.
